# The nature and contribution of innovative health financing mechanisms in the World Health Organization African region: A scoping review

**DOI:** 10.7189/jogh.13.04153

**Published:** 2023-11-15

**Authors:** Juliet Nabyonga-Orem, Christmal D Christmals, Kingsley F Addai, Kasonde Mwinga, Diane Karenzi-Muhongerwa, Sylvia Namuli, James A Asamani

**Affiliations:** 1 World Health Organization Regional office for Africa, Office of the Regional Director, Brazzaville, Congo; 2 Centre for Health Professions Education, Faculty of Health Sciences, North-West University, South Africa; 3 World Health Organization Ghana country office, Universal Health Coverage Life Course Cluster, Accra, Ghana; 4 World Health Organization, Africa regional office, Universal Health Coverage Life Course Cluster, Brazzaville Congo

## Abstract

**Background:**

Achieving financial risk protection for the whole population requires significant financing for health. Health systems in low- and middle-income countries (LMIC) are plagued with persistent underfunding, and recent reductions in official development assistance have been registered. To create fiscal space for health, the pursuit of efficiency gains and exploring innovative health financing for health seem attractive. This paper sought to synthesize available evidence on the nature of innovative health financing instruments, mechanisms and policies implemented in Africa. We further reviewed the factors that hinder or facilitate implementation, the lessons learnt on the structure, the development process and the implementation.

**Methods:**

We conducted a systematic scoping review of the literature to analyze the nature, type, and factors impacting the implementation of innovative health financing mechanisms in the World Health Organization (WHO) African region.

**Results:**

Innovative health financing mechanisms are increasing in the WHO African region as a result of international policy, the need to improve healthy eating and social life of the populace, advocacy and the availability of international mechanisms to which countries can subscribe. The 41 documents included in this review reported ten innovative financing mechanisms in 43 out of the 47 WHO Africa region member states. The most common mechanisms include an excise tax on tobacco products (43 countries) and alcoholic beverages and spirits (41 countries), airline ticket levy (18 countries), sugar-based beverages tax (seven countries), and levy on oil, gas and mineral tax (four countries). Other mechanisms include the human immunodeficiency virus/acquired immunodeficiency syndrome (HIV/AIDS) trust fund, the social impact bond, the financial transaction tax, mobile phone tax and equity funds. Funds generated from many mechanisms are not allocated to health, although some portions are allocated to health-related activities. In some countries where mechanisms implemented are public health-related, emphasis is placed on positive health behavior beyond raising funds. Persistent resistance from industries due to conflicting economic policies is a major challenge.

**Conclusions:**

Leveraging international policies and setting up intersectoral committees to develop and implement innovative mechanisms that involve excise taxes are recommended as possible solutions to the conflicts of interest.

Financing for health is a critical component in the universal health coverage (UHC) journey, the lack of which is a major concern, as stated by Suborna [1]. We learn lessons from failed attempts to meet the millennium development goals, especially in poor countries. Insufficient amounts, short-term horizon of donor aid, and fragmented and misaligned resource allocation patterns characterized the health financing landscape in poor countries [2]. Perhaps this explains the change in emphasis as the world embraced the sustainable development goals (SDGs). The SDGs envisaged governments playing a significant role in funding the 2030 agenda and the UHC aspirations. However, in the past ten years, the relative importance that African governments have accorded to health has stagnated at a regional average of just slightly over 30% of current health expenditure [3]. In the World Health Organization (WHO) African region, 35 countries are spending less than the minimum required to ensure access to the essential health package of services estimated at US$112 per capita, compared to the regional average of US$124 per capita [4].

Official Development Assistance (ODA) through bilateral and multilateral mechanisms has been declining since 2013 [5] and is projected to be further adversely affected by the coronavirus disease 2019 (COVID-19) pandemic shocks. Although financial protection to ensure that no persons are excluded from accessing health services or are impoverished by the high cost of health care is the cardinal tenet of UHC, it has implications for increasing health expenditure [6,7]. Additionally, the population’s need for health care is ever-increasing due to increasing life expectancy and advancements in medical technology hence, health expenditures are expected to escalate yearly [8-10]. For example, it has been projected that in addition to current levels of health expenditure, US$274 to US$371 billion is needed annually until 2030 to achieve the health system targets in the SDGs [11,12]. Most countries in the African region, however, have limited domestic revenue-raising capacity, while the budgetary allocation or priority given to health leaves much to be desired. For instance, only two countries met the Abuja target of allocating 15% of the government budget to health in 2020 [13]. In the context of economic shocks experienced by countries due to the protracted COVID-19 pandemic, government revenues have declined, and the fiscal space is expected to shrink in the short to medium term [11,12].

Building resilient health systems that ensure health security and universal health coverage within a limited fiscal space calls for improving health financing. The health financing policy discourse identifies improving efficiency and exploring innovative health financing mechanisms as plausible solutions to the challenge. Healthcare financing covers the mobilisation, pooling and equitable allocation of funds in the health system in a manner that allows the population to receive quality and safe care without being pushed into poverty as a consequence of health care costs [14]. Traditionally, there are three broad health care financing models. The Bismarck and Beveridge systems include social insurance through employment, national health insurance in all forms, and the out-of-pocket model. Being innovative goes beyond merely modifying the traditional funding mechanisms.

The WHO task force defined innovative financing as a range of non-traditional mechanisms designed to raise additional funds for health through innovative projects such as micro-contributions, taxes, public-private partnerships and market-based financial transactions [15,16].

A financing mechanism must meet the essential criteria to be innovative. The first criterion is additionality, meaning the innovative financing instruments, mechanisms or policies are created to fill the financial gaps that have compromised the attainment of health targets. Thus, innovative financing mechanisms must complement existing funding and not substitute them or have a crowding-out effect on pre-existing budgetary commitment. The second criterion is effectiveness, meaning the mechanism ensures the right and better use of the additional funds, and the third criterion is efficiency in ensuring value for the use of the additional funding from innovative financing mechanisms, instruments, or policy, ensuring transparency and accountability.

Countries in the African region have initiated various forms of innovative financing for health, including the so-called sin taxes (on sugar, alcohol, cigarettes and other tobacco products), mobile phone or communication tax, HIV/AIDS levies, tax on insurance and other instruments of micro-contributions to fund specific aspects of health care. For example, Rwanda’s innovative financing model [17] has been seen as one similar to the HIV/AIDS levy in Zimbabwe, 50% of funds collected subsidise antiretroviral treatment [18]. Although several countries have innovative financing mechanisms, certain lingering issues must be addressed to facilitate peer-to-peer learning toward the adoption and/or improvement of innovative health financing mechanisms for sustainable health financing. We analyzed available evidence on the nature of innovative health financing instruments, mechanisms and policies implemented in Africa. We further reviewed the factors that hinder or facilitate implementation, the lessons learnt on the structure, the development process and the implementation.

## METHODS

We conducted a systematic scoping review [19,20] covering the period from 2010 to 2022 to describe the innovative health financing mechanisms in the WHO African region. Scoping reviews are undertaken as stand-alone or pre-systematic reviews [21]. The underlying motivation for scoping reviews is that the area under study is emerging and not well-researched or very broad to focus a focused review. Innovative health financing mechanisms are an emerging area, especially in the WHO African region, and scoping reviews permit the inclusion and synthesis of studies from all paradigms [22,23]. To successfully synthesize all the concepts related to innovative financing mechanisms in the WHO African region, we included government policy documents, reports and qualitative, quantitative, mixed-method and multi-method published studies on the phenomenon [22,23]. This requires a review method that is broad and inclusive enough to achieve the purpose sought [24]. We conducted a convergent integrated synthesis and integration of qualitative and quantitative findings from included studies [25]. We followed Arksey and O’Malley’s [26] scoping review framework, comprising of six phases – identifying the research question, identifying relevant studies, study selection, charting the data, collating, summarizing and reporting the results, and consultation (optional).

### Identifying the research question

The review question considered in this study was guided by the population, concept and context (PCC) mnemonic [23]. Regarding population, we included health financing for all population groups in the WHO African region. The concept included the nature, structure, facilitators and inhibitors, lessons from the development process and implementation of innovative health financing instruments and policies. Further, the context was related to the 47 countries in the WHO African region.

### Identifying relevant studies

We conducted search in four phases: database search, forward and backward search, snowballing through experts, and hand search. Phase one involved a systematic computerised search in PubMed, Scopus, Web of Science, EbscoHost and ProQuest, using various combinations of the keywords (**Table 1**). The search string and the link to the results of an advanced search are presented in the **Online Supplementary Document****.**

**Table 1:** Keywords and their substitutes

Keywords	Alternative words used
Innovative financing	Sin taxes
Domestic revenue	Tax on car insurance for health
	Sugar tax for health
	Salt tax for health
	Mobile phone tax for health
	Airline ticket tax for health
	Cigarette tax for health
Health	Health care
	Health services
	Health systems
Africa	Countries within the WHO African region*

WHO – World Health Organization

*Algeria, Angola, Benin, Botswana, Burkina Faso, Burundi, Cabo Verde, Cameroon, Central African Republic, Chad, Comoros, Congo, Côte d'Ivoire, Democratic Republic of the Congo, Equatorial Guinea, Eritrea, Eswatini, Ethiopia, Gabon, Gambia, Ghana, Guinea, Guinea Bissau, Kenya, Lesotho, Liberia, Madagascar, Malawi, Mali, Mauritania, Mauritius, Mozambique, Namibia, Niger Nigeria, Rwanda, Sao Tome and Principe, Senegal, Seychelles, Sierra Leone, South Africa, South Sudan, Togo, Uganda, United Republic of Tanzania, Zambia, Zimbabwe.

Phase two comprised forward and backward or ancestry searches on the relevant studies. Forward search includes searching for papers that cite a specific study, while backward search involves searching through the reference list of a paper to retrieve relevant studies. In phase three, all corresponding authors of the included studies were contacted to lead the researchers to papers and other reports on innovative financing in the WHO African region that the authors might have missed. The Google Scholar, ORCID and ResearchGate accounts of the authors of relevant studies were scanned. Also, websites of relevant institutions such as WHO, African Union, World Bank, and ministries of health in the WHO African region were hand-searched for relevant policy documents on innovative health care financing. Finally, health financing experts in the WHO African region country offices were contacted to facilitate identifying and collecting relevant documents and reports on innovative health financing within the African region.

### Study selection

We set the inclusion criteria to identify studies, reports, or policy documents published on any form of innovative health care financing mechanisms. Further, the search included peer-reviewed and gray literature (editorials, commentaries, research, analysis, opinions, reports, etc.). No year limits were set on studies to be included in order to trace the genesis of the innovative financing mechanisms. Further, there were no language restrictions. Regarding the exclusion criteria, the search excluded studies published about countries outside the WHO African region, studies published on traditional health financing mechanisms (studies that do not meet the three essential criteria of innovative financing mechanisms), and studies that do not mention the country (setting).

### Selection of eligible studies

We imported all identified studies into a Mendeley (1.19.8, Mendeley Ltd, Elsevier, Amsterdam, Netherlands) reference manager to identify and merge duplicates. Further, we screened titles, followed by screening the abstracts to exclude studies that did not fit the scope of the review. Full-text articles of relevant studies were included for further evaluation, ie, critical appraisal. Three reviewers independently selected studies, and potential discrepancies regarding the inclusion or exclusion criteria were resolved by consensus between the three reviewers. The quality appraisal of the studies was not performed as policy documents and peer-reviewed papers of any kind were included in this review. Data from different sources were used to validate each other to ensure the accuracy of the content presented.

### Charting the data

We retrieved relevant findings from the included studies and documents that mentioned innovative financing mechanisms in Africa and extracted into a data matrix highlighting the financing mechanism/ instrument, year of inception, setting /country, nature of the document (scientific publication, policy, report act, etc.), facilitators, inhibitors, nature/ structure, lessons from development and implementation processes and whether or not earmarked for health (Table S1 in the **Online Supplementary Document**). Where there were conflicting values, we retrieved the most recent data. Preference was given to authentic, country-specific data over global reports in cases where different values were reported. For example, the WHO report on the global tobacco epidemic 2019 [27] reported a 33.3% excise tax on tobacco in Ghana as against the 175% reported by the parliament of Ghana [28] in the Excise Duty Amendment Act gazetted. Therefore, we reported the figures from the parliament of Ghana [28].

### Collating, summarizing and reporting the results

We conducted a convergent integrated synthesis and integration of qualitative and quantitative findings from the included studies [22,23]. We transformed results from quantitative studies, screened for quality, and synthesized qualitatively with the findings from the qualitative studies [22]. Assessing the quality of quantitative findings involved narratively describing numbers and figures to present a qualitative representation or translation of such findings without changing their value [22]. We examined the textual (qualitative) data, pooled into categories and described similarly to the meta-aggregation process of qualitative studies [22]. We report findings according to the Preferred Reporting Items for Systematic Reviews and Meta-Analyses extension for Scoping Reviews (PRISMA-ScR) guidelines [29].

## RESULTS

### Characteristics of studies

Our database search yielded 171 journal papers, of which 15 were included [30-44]. Five of the empirical studies on innovative financing mechanisms were from South Africa [30-34], five studies were intercountry [35-39], two from Zambia [40,41], and one each from Botswana [42], Mauritius [43] and Uganda [44].

**Figure 1:**
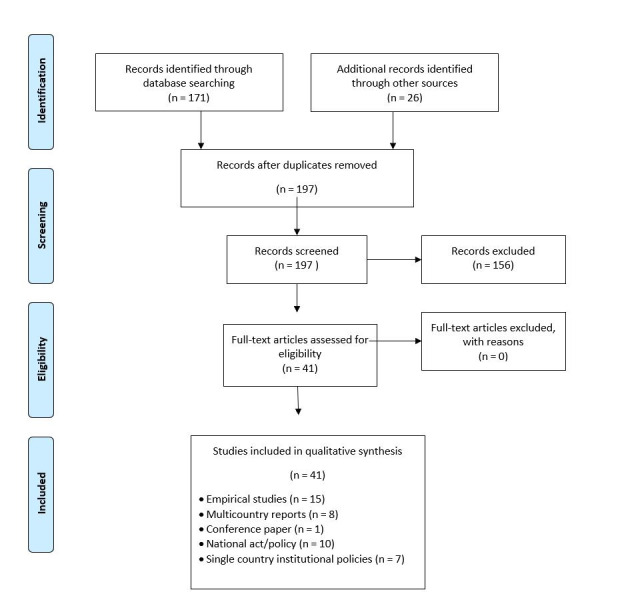
Search and inclusion of studies.

Regarding the gray literature, we identified 26 documents, comprising ten national acts/policies [28,45-53], eight multi-country reports [27,54-60], seven single-country institutional policies [61-67] and a conference paper [68] (**Figure 1**). Some studies reported one mechanism, while others reported more than five. The WHO produced the majority of reports (n = 6) [27,54,57,60,65].

### Innovative financing mechanisms available

Documents included in this review (n = 41) reported ten innovative financing mechanisms in 43 of the 47 WHO African region member states (**Table 2**). The most common mechanisms included excise taxes on tobacco products (43 countries), excise tax on alcoholic beverages and spirits (41 countries), airline ticket levy (18 countries), sugar-based beverages tax (seven countries), and levy on oil gas and mineral tax (four countries). The rest of the mechanisms include an HIV/AIDS trust fund, social impact bond, financial transaction tax, mobile phone tax, and equity fund. It is worth noting that not all the mechanisms generate additional money for the ministries of health, but all the mechanisms, in one way or another, improve health care or health behavior. Loan buydown and Debt2Health were excluded as innovative mechanisms, although they generate extra funds for the health system. We did not consider debt relief as innovative.

**Table 2:** Summary of identified innovative health financing mechanisms

Country	Tobacco tax	Alcohol tax	Airline levy	Sugar tax	Oil, gas, minerals	HIV/AIDS trust fund	Social impact bond	Financial transactions tax	Mobile phone tax	Equity fund
Algeria	X									
Benin	X	X	X							
Botswana	X	X								
Burkina Faso	X	X	X							
Burundi	X	X								
Cabo Verde	X	X								
Cameroon	X	X	X							
Central African Republic	X	X	X							
Chad	X	X								
Comoros	X	X								
Congo	X	X			X					
Côte d’Ivoire	X	X	X							
Democratic Republic of the Congo	X	X	X							
Equatorial Guinea	X	X								
Eritria	X	X								
Eswatini	X	X								
Ethiopia	X	X								
Gabon	X	X	X					X	X	
Gambia	X	X								
Ghana	X	X								
Guinea	X	X	X		X					
Kenya	X	X		X						
Lesotho	X	X								
Liberia	X	X	X							
Madagascar	X	X	X							
Malawi	X	X								
Mali	X	X	X		X					
Mauritania	X	X								
Mauritius	X		X	X						
Namibia	X	X	X							
Niger	X	X	X		X					
Nigeria	X	X								X
Rwanda	X	X		X						
Sao Tome and Principe	X	X	X							
Senegal	X	X	X							
Seychelles	X	X								
Sierra Leone	X	X								
South Africa	X	X	X	X			X			
Togo	X	X	X							
Uganda	X	X		X		X				
United Republic of Tanzania	X	X		X						
Zambia	X	X		X						
Zimbabwe	X	X				X				

AIDS – acquired immunodeficiency syndrome, HIV – human immunodeficiency virus, X – available

### Nature of innovative financing mechanism implemented in the WHO African region

The nature of the innovative health financing mechanisms implemented on the continent was described in terms of the structure, the mode of collection and the allocation of the resources. The innovative health financing mechanisms in the WHO African region can be classified into four main categories – sin taxes, levies and bonds, transaction taxes, and trust funds and equities.

### Sin taxes (excise taxes on harmful goods)

Many countries in the WHO African region imposed sin taxes on selected goods that were perceived as harmful to the health of the population. These goods include tobacco [69] and cigars, which are proven predisposing factors to many cardiovascular, respiratory and digestive disorders. Also, alcoholic beverages, which predispose the population to non-communicable diseases, injuries and violence, have been taxed by many countries in the WHO African region [70]. Excise taxes on sugar-sweetened beverages are the most discussed sin tax in the last five years, with countries evaluating its feasibility and developing policies to raise funds for health, sports and recreational activities [33,40].

Sin taxes are voluntary tax systems, paid only when the product or services are used. They are either ad valorem, that is, taxing according to the value of the goods, as seen in many of the excise taxes on the continent [27] or specific values imposed on the goods. In most cases, it is charged as a percentage of the goods' factory or retail price, as seen in Mauritius, where a 183.5% excise tax is imposed on cigarettes [27]. Other instruments also charge specific amounts per package or weight of goods without segregating them according to the percentage composition of the harmful substance. For example, the Malawi Revenue Authority [51] imposed US$15 per 1000 cigarette sticks. Lastly, a combination of ad valorem and fixed taxes is used in the region [51]. An example of the combination tax regime is observed in Zimbabwe, where an excise tax of 30% is charged on the retail price of alcoholic beverages plus 10 Zimbabwe dollars per liter, and also in Cabo Verde, where 30 to 50% tax is imposed on cigarettes, cigars and waterpipe tobacco plus 20$00 escudos on each packet of cigarettes [68,71]. Sin taxes are usually imposed in addition to other regulatory mechanisms to cause a change in the social use of or dependence on the goods that are considered harmful to the life of the population. Time, age, place of use, and percentage composition restrictions are standard policies associated with sin taxes.

### Excise tax on tobacco

Regarding excise taxes on tobacco products (cigarettes, cigars, waterpipes), Uganda, Ghana, and Mauritius impose the highest percentage (200,175, and 84%). Countries with the least percentage tax are Mauritania (9.6% excise tax on tobacco), Guinea-Bissau (6.8% excise tax on tobacco) and Benin (4.9% excise tax on tobacco).

Globally, almost all countries impose excise taxes on tobacco products. In some countries, however, an additional percentage was added to the excise taxes or the already existing taxes were repurposed to fill a health care gap. The excise taxes in these two categories are termed innovative health financing. In the WHO African region, Algeria, Botswana, Cabo Verde, Chad, Comoros, Congo and Mauritania have either repurposed or added to the existing excise tax on tobacco for health or recreation (**Table 3**). For example, in Algeria, an additional excise tax component is invested in emergency care services. Chad and Mauritania invest 2%-7% of the excise tax in antiretroviral medications and cancer research, while in Congo, half of the money raised is invested in health insurance [69,72]. Comoros invests all the money generated from the tax on tobacco in sports and emergency care, while Cabo Verde invests all the money raised from the tobacco tax in sports and health [72]. In Ethiopia, the Ministry of Health has synthesized research evidence to motivate the money gained from tobacco taxes to be invested in health care [45,72]. Also, research has shown that in Gambia, Madagascar, Mauritius, Mozambique, Senegal, Uganda and Zimbabwe, the increasing tax on tobacco products has made them less affordable, thereby curbing their use [27].

**Table 3:** Tobacco tax allocated to health and health-related activities in Africa*

Country	Tobacco tax system in %	Earmarked for health
Algeria	34.2	Used for emergency health services.
Botswana	49.9†	Earmarked for health.
Cabo Verde	11.2	Used for sports and health care.
Chad	34.1	An additional 2% tax is used for antiretroviral medication.
Comoros	37.3	A portion of the 5% extra tax on tobacco is directed to the Ministry of Sports and another portion to hospital emergency services.
Congo	38.7	50% allocated to health insurance and the other half for sports.
Côte d’Ivoire	33.3	2% of producer prices are used for HIV/AIDS care.
Madagascar	80.4	Allocated to tobacco control, sports and culture
Mauritania	9.6	Extra 7% on import costs earmarked for cancer research.
Mauritius	83.5	Portion of the funds allocated for the treatment of tobacco-related conditions.

AIDS – acquired immunodeficiency syndrome, HIV – human immunodeficiency virus

*Adapted from WHO report on the global tobacco epidemic 2019: offer help to quit tobacco use [27] and use of earmarked tobacco taxes [72].

†On production cost and import cost.

### Excise tax on alcohol

The quantum of excise taxes on all forms of alcohol vary considerably across the WHO African region. Taxes are imposed on alcohol in 41 out of the 47 countries in the WHO African region (**Table 3**). The rates vary in the specific amount set on a liter of liquor, wine and spirits with different strengths of alcohol content. In South Africa, US$4.88 is charged per liter of spirit, US$2.89 is charged per liter of beer and US$0.49 per liter of traditional beer. Furthermore, 3.7 South Africa Rands is charged per liter of fortified wine and 6.2 per liter of sparkling wine. In Benin, Botswana, Cabo Verde, Ghana and Zambia, the ad valorem tax rate is applied. Botswana started with a 30% excise tax on alcoholic beverages in 2008. The percentage increased to 55% in 2015. Cabo Verde also implements a percentage excise tax of 50% on all alcoholic drinks and spirits. In Zambia, a 35%-75% excise tax was imposed on all alcoholic beverages but reduced to 60% in 2009 and 40% in 2011. Ghana set 30% while Benin takes only 10% of the value of alcoholic drinks in tax. In Botswana, 55% of the retail price of alcoholic products is imposed as tax, of which 10% is allocated to the Ministry of health, 45% to the consolidated fund and 45% to the Ministry of youth [42,55]. An additional 2% is, however, specifically taxed and allocated as the HIV trust fund, which is another form of the innovative financing mechanism. Ethiopia’s Ministry of health advocates for the funds raised from taxes on alcohol to be invested in health care [45].

### Excise tax on sugar-based drinks

Eight countries in the WHO African region – Kenya [37], Mauritius [43], Nigeria [52], Rwanda [37], South Africa [31-33,53], Uganda [37], United Republic of Tanzania [52], and Zambia [38,40,41] – have implemented excise taxes on sugar-sweetened beverages.

In Mauritius, a specific excise tax of six cents per gram of sugar is imposed on the industry. To motivate economic activity, drinks for export are exempted from tax. This is to reduce sugar consumption, a well-established factor linked to obesity and its associated non-communicable diseases and diabetes in Mauritius [43]. In South Africa, the first four grams (per every 100 mL) of sugar-sweetened drinks are not taxed. Subsequent grams are charged per the prevailing rate of 2.1 South African cents per gram. The tax is charged at the factory. This constitutes US$0.15 per gram of sugar in sugar-sweetened beverages. This was done under the Health Promotion Levy (HPL) to reduce the high intake of sugar-sweetened drinks with their associated health risks. This led to an 11% increase in the cost of sugar-sweetened beverages in South Africa [31-33,53]. The rate is adjusted yearly to cater for inflation. In Zambia, a 25% excise tax on sugar-sweetened beverages was introduced in 1998. This was repealed in 2015 to favor economic policies and reintroduced in 2018 at 3% on imported items and 0.5% on locally manufactured goods [37,38].

Uganda earmarks all the funds generated from tax on sugar-sweetened drinks for their HIV/AIDS services under the HIV trust fund [37]. Rwanda and the United Republic of Tanzania also earmark those funds for health care. In South Africa, a portion of the revenue generated is earmarked for health promotion activities across the country [33,53].

### Levies and bonds

This class of innovative health financing mechanisms comprises levies charged on air tickets [55,59], commitments from governments to invest portions of the funds generated from minerals mined in their countries in health care [59], and payment of the total cost of health care projects undertaken by local institutions funded by other organisations if the project delivers on the set objectives [34,66].

### Airline ticket levy

This is an innovative mechanism implemented by a third party, Tous Unis pour Aider (UNITAID) on behalf of interested countries. In the WHO African region, 19 countries – Benin, Burkina Faso, Cameroon, Central African Republic, the Democratic Republic of the Congo, Côte d’Ivoire, Gabon, Guinea, Liberia, Madagascar, Mali, Mauritius, Morocco, Namibia, Niger, Sao Tome and Principe, Senegal, South Africa and Togo – have all subscribed to the mechanism. The airline charges a mandatory solidarity levy of US$1 on the economy and US$10 on business class airline tickets. Although the mechanism is initiated at the country level, it is coordinated by UNITAID, while the airlines collect the levies. Funds collected from the airlines are transferred to the national treasury by UNITAID to fund programmes for AIDS, tuberculosis and malaria in developing countries.

### Micro levy on oil, gas, gold and other mining activities

Micro levy on oil, gas, gold and other mining activities is managed like the airline ticket levy. This mechanism is called a levy as it involves the voluntary subscription of organisations to provide additional funding for specific sector development. It is sometimes referred to as a solidarity levy. A third-party organization, the United Nations Trust Fund to Prevent Chronic Malnutrition (UNITLIFE), initiated this mechanism to which countries subscribe. UNITLIFE is a unit set up by United Nations (UN) Women, the UN Capital Development Fund (UNCDF), the government of France, and the Abu Dhabi Crown Prince Court as an innovative method of raising funds to fight malnutrition during the first 1000 days of a child’s life. With this mechanism, 0.1% of mining, gold, oil and gas revenues are deposited into the UNITLIFE funds by any country that subscribes to the mechanism and used to fight malnutrition in the countries that have subscribed to the mechanisms [59].

### Social impact bond

The social impact bond is a three-year mechanism implemented by the South African Medical Research Council as an HIV prevention intervention for adolescent girls and young women (AGYW) [34,66]. The Global Fund funded the social impact bond from 2019 to 2022, with the agreement that the South African government would pay the money back to the Global Fund if the South African Medical Research Council delivered services on behalf of the government when the outcomes have been achieved.

### Transaction taxes

These are taxes imposed on financial transactions to raise funds for health care. Two types of transaction taxes were reported in the WHO African region – mobile phone tax [57,60,65] and financial transactions tax [57,60,65] reported by Gabon. There was also a 1.5% additional levy on profit after tax on currency and other transactions. The tax was paid by companies that handle remittances. Just like the financial transactions tax, Gabon implemented the mobile phone tax. The instrument involved a 10% tax on mobile phone operators. The funds raised from both mechanisms are earmarked for subsidising health care for the poor in Gabon. It covers more than 99% of the poor.

### Trust funds and equity funds

Trust funds and equity comprise the mechanisms that seek to harmonise funds generated from donations by a section of the population [67] from government allocation [39,44,64] to provide care for a group of people who need it.

The HIV/AIDS trust fund is an innovative financing mechanism employed by Uganda and Zimbabwe. In Uganda, the trust fund was established by an Act of Parliament, imposing a 2% tax on alcoholic beverages, soft drinks and bottled water to fund HIV care. Although the source of funding for this mechanism (tax on alcoholic beverages and soft drinks) could fall into other categories of innovative financing, this mechanism is holistically considered a trust fund. It was projected to raise about US$2 million annually, covering about 0.5% of the total annual expenditure on HIV/AIDS care [44].

In Zimbabwe, the National AIDS Council of Zimbabwe Act added a 3% levy to the Pay-as-you-earn (PAYE) tax and a 3% income tax for corporate bodies to tackle the burden of HIV/AIDS in Zimbabwe. It is estimated that about US$35.5 million was raised in 2014, covering about 15% of expenditure on HIV/AIDS care in Zimbabwe [39,55].

### Other mechanisms

We also found that other mechanisms, such as Debt2Health and loan buydown, generate an additional funding source for the health system. Debt2Health is a debt relief program where a creditor forgives a debtor a portion of the loan to invest in health. For example, Germany forgives Côte d’Ivoire a debt of US$27 million if Côte d’Ivoire invests at least 50% of the debt in HIV/AIDS programmes [39]. On the other hand, a loan buydown is a mechanism in which a debtor is forgiven part or all of the loan facility they received if they meet the set objectives of the project for which the loan was credited. Sometimes, a donor may commit to paying back the loan if the debtor meets the project objectives. An example is the buydown mechanism that occurred in Botswana, where US$50 million was granted to the country by the World Bank, with the European Commission committing to pay (buydown) US$20 million of the loan received if Botswana meets specific objectives. These two debt conversion mechanisms were not considered innovative health financing, although other institutions, such as the Global Fund [55], ascribe that title to them [7].

### Factors facilitating or inhibiting the implementation of innovative financing mechanisms

#### Facilitating factors

The need to improve lifestyles and population health has been pivotal in the implementation of innovative health financing mechanisms. Impact on the health of individuals, curbing indiscriminate/unprotected sexual intercourse and associated increased HIV incidence rates and the rising burden of accidents and injuries in most parts of Africa are some of the factors that have provided the impetus for imposing an excise tax on alcoholic beverages [35,42,54]. Again, the consumption of alcohol has been associated with social vices such as gender-based violence [42]. Turning to excise tax on sugar-sweetened beverages, the growing burden of non-communicable diseases generally forms the foundation of the policy in all countries.

International instruments and policies were beneficial in some cases. Turning to tobacco taxes, the highest influencing factor is the international policy on tobacco, such as the WHO Framework Convention on Tobacco Control (FCTC) [73] and Economic Community of West African States ECOWAS tax [63]. Countries are working to meet the requirements of this policy by adjusting the excise taxes to reach the required 70% of the retail price of tobacco products. Multisectoral policy formulation and political will were also cited as facilitating factors [63,74]. Regarding the excise tax on sugar-sweetened beverages, the WHO fiscal policies for diet and prevention of non-communicable diseases influenced the sugar-sweetened beverages tax policies [74].

Minimal impact on the industry and ease of administration have facilitated the implementation of air ticket levies and micro levies. These taxes have been scientifically proven to have no influence on air traffic. The instruction is clear, transparent, cost-effective to generate, and acceptable to contributors – the levy is relatively small compared to the airline ticket cost.

The credibility of the managers has been beneficial in the case of a social impact bond. For example, the executing institution (South African Medical Research Council) has a good track record and credibility in managing the fund, hence, it was trusted by both the funder (the Global Fund) and the government. There was a government commitment to pay the funder according to agreed objectives, although there was little or no government interference during the project execution.

Key factors that facilitated the success of the HIV trust fund in Zimbabwe include advocacy by people living with HIV, establishment of the fund as a seed fund to attract donor funding, strong cooperative governance, clearly defined use of funds, annual work plan, and budget, fund administered according to laid down policies, fund decentralised to the lowest level and active participation of district AIDS action committees. In Uganda, the HIV fund is an instrument that seems to have stemmed from strong political will.

The mode of collection also impacted innovative financing mechanisms. The mobile phone tax was also easy to implement and did not attract any public resistance because it was directly collected from the telecommunication companies rather than the populace.

#### Inhibitory factors

Several factors negatively impacted the implementation of innovative financing mechanisms. Resistance from both the industry and the population in the case of the tobacco, sweetened beverages and alcohol industries and the economic impact of levying taxes played a negative role. This included using economic blackmail to pressure the government to reduce or scrap the sin tax on alcohol. A typical example was observed in Zambia, where the sin tax on alcoholic beverages decreased from 75 to 60% in 2009 and 40% in 2015 to favor the growth in the alcohol industry. Second is the resistance from the population that uses alcohol [42], many of whom tried to make the government unpopular for waging war against alcohol users. They purchase alcohol across the border from other countries which do not have the same regulations.

The strongest resistance stemmed from the government’s economic policies that conflicted with the sin taxes. For example, the government’s projected growth in the alcohol industry is hampered by the taxation imposed on the industry [75]. Conflict with economic policies and lobbying from the tobacco industry are key factors hindering higher tobacco product taxes [59]. Similarly, although the financial transaction taxes were easy to implement and provided quick funds for the health sector, the population resisted the instrument, citing the negative influence of the instrument on the national economy as the reason [59].

The suboptimal consultation process is another hindrance. For example, although air ticket levies are acceptable, stakeholder consultation is poor as no organized traveler grouping could be consulted. Also, they are deliberated at high political levels and are very slow in implementation [75].

Specific to transaction taxes, challenges to the implementation of the instrument included delays due to bureaucracy, lack of clarity in policy, probability of not using funds for HIV if clearly defined roles are not imputed, coverage of only 0.5% of HIV expenditure, and insufficient technical support for the policy in Uganda [44,64]. Regarding Zimbabwe, although about 70% of the population employed in the informal sector was covered by the fund, high inflation rates eroded the value of funds collected [55].

#### Use of collected funds

In Botswana, 90% of the HIV/AIDS funds were used to procure antiretroviral medications, leaving just a little for health promotion and preventive activities [42]. The government, therefore, needed more funding to invest in HIV prevention activities. Also, lifelong Antiretroviral therapy (ART) treatment for people living with HIV requires sustainable local financing [42].

### Lessons learnt on the design (structure), development and implementation process

#### Influence of international policies and innovative financing mechanisms

International health policy is a critical influencer in the conceptualisation, development and implementation of innovative health financing mechanisms. Article six of the FCTC commits parties to use price and tax measures, including excise taxes on tobacco products to reduce tobacco use. Many countries aspire to meet WHO’s 70% excise tax threshold. Some countries have imposed higher than the threshold, and research has proven that setting higher taxes reduces the harmful products’ affordability, thereby reducing their use [63,68,73].

The availability of international mechanisms that countries could subscribe to with minimum effort and less administrative involvement also influenced the implementation of innovative health financing mechanisms in the WHO African region. The airline ticket levy, conceptualised and coordinated by UNITAID to fund tuberculosis, malaria, HIV/AIDS and associated infections, is a way of influencing countries to subscribe to the mechanism [7,76]. This is also true for the micro levy on oil, gas, gold and other mining activities introduced by UNITLIFE to prevent malnutrition in the first 1000 days of life [59]. These mechanisms are easy to manage and administer. The funds are used for the purpose they are raised for. International instruments such as the levies coordinated by UNITAID and UNITLIFE seem compelling.

#### Implementation of the mechanism in a cluster

Sin taxes seem to be successful when implemented as part of a cluster of mechanisms to improve the health of the population. In addition to appropriate excise taxation policies, there is a need for complementary non-tax measures that can be effectively targeted at specific consumers and high-risk behavior patterns. Educational programmes and regulatory interventions to discourage risky and hazardous consumption of unhealthy goods are necessary to complement the excise tax regime [41,43,68].

#### Intersectoral collaboration is essential for acceptability

Most of the resistance related to sin taxes emanates from the ministries of finance. They also have the mandate to increase growth in the tobacco, alcohol and sugar industries, in addition to lobbying the big players in the product market [33,37,38]. An intersectoral tax policy that considers the necessary national economic programmes and health-related goals is essential to the success of the sin tax mechanisms. Broad-based stakeholder consultations and education on the policy documents required prior to implementation is also critical to the smooth performance of the policy [33,37,38].

#### Country-level commitment attracts funding

It can be deduced that countries making a sustainable commitment to health care have the potential to attract donor funding. The Zimbabwean aids trust fund is considered a best practice in Africa. It provided the national AIDS council resilience through economic recession [39,55]. Compared to Zimbabwe, the Zambian trust has policy challenges. Koseki et al. [64] and Birungi and Colbourn [44] revealed that although the policy provides a sustainable funding source, there are questions regarding stakeholder involvement and the structure of the trust fund. It was also reported that the drive for the trust fund was more political than technical [44].

#### Advocacy by the ministries/departments of health and social groups

In Zimbabwe, advocacy by the population in need played a crucial role in the sourcing of funds for the HIV trust fund. The evidence-based investment case made by the Ethiopian Ministry of Health [45] was also influential in implementing the excise tax on sugar-sweetened beverages. Research had shown that healthy eating behaviors were observed when an excise tax on sugar-sweetened drinks was implemented. For example, in South Africa, empirical research has shown that households in urban communities reduced sugar-sweetened drinks consumption by half. Also, compared to non-taxable beverages, consumer purchases of taxable beverages increased significantly after the policy was implemented. The threshold approach in South Africa incentivises manufacturers to shift to lower-sugar beverages to avoid tax. Academic publications and advocacy by educational institutions were also effective in furthering the implementation of sin taxes in South Africa [32,40,77]. In Zambia, the alcohol tax was lobbied for by the Ministry of Health [40].

#### Transparent management of funds builds donor confidence and local commitment

It was reported that the transparent use of the HIV trust fund in Zimbabwe built contributor and donor confidence in the instrument. The commission is responsible for managing the funds accounts to the population through media updates, generating trust in the trust fund. UNITAID and UNITLIFE levies were also transparently managed, building country confidence in such mechanisms [7].

#### Need for ministries of health to push for conversion of existing mechanisms into health financing mechanisms

There are excise taxes on tobacco, sugar and alcohol in almost all countries in the WHO African region, but not all countries earmark a portion or all of these taxes for health care. Knowing the adverse effect of these goods on the population’s health, the ministries of health can use evidence to make a case for earmarking such revenues for the health sector.

#### Data are essential in evaluating the impact of mechanisms

Data challenges marred the evaluation of the impact of the social impact bond implemented in South Africa. It was also reported that coverage was poor in the first year of implementation as COVID-19 lockdown and school closures affected coverage. It is essential to set up empirical studies to collect data on the effectiveness of innovative health financing mechanisms for evaluation. South Africa has successfully evaluated the impact of the excise tax on sugar-sweetened beverages [32,40,77].

## DISCUSSION

Innovative health financing is growing on the continent and the funds are being reviewed in terms of health policy. Ministries of health are also beginning to receive some funds from the proceeds as well as benefits of the mechanisms. For example, most countries that implement a tobacco tax (excise tax on all tobacco products) automatically cause an increase in the price of tobacco products, thereby reducing their affordability and use. However, the tax generated goes to the consolidated fund in the majority of cases. The same observation is made for taxes imposed on sugar-sweetened beverages and alcohol products. One difficulty is drawing the line between health financing and other forms of financing. Is funding sports and recreation considered to be health financing? To what extent is funding not allocated to the ministry of health defined as health financing?

The growing trend in innovative health financing mechanisms across the continent could be attributed to four broad factors. First, international policies on harmful products, such as the WHO FCTC, influence excise taxes on tobacco products [73]. The WHO fiscal policies for diet and prevention of non-communicable diseases [74] also significantly impact the imposition of excise taxes on sugar-sweetened drinks across Africa. The second is the availability of international mechanisms to which countries could easily subscribe. These international mechanisms include the airline ticket levy managed by UNITAID and the levies on oil, gas, gold and other mining activities collected by UNITLIFE [7]. Third, learning from each other, many countries are considering introducing an excise tax on sugar-sweetened beverages because it has been successfully introduced in other countries [37]. This could also be attributed to the publicity received by these policies on the continent. Situational analysis and recommendations have been made in many countries. Ministers of health need to learn from the example of the Zambia Ministry of Health to advocate for such sin taxes to be allocated to health, if they already exist, or if otherwise imposed [78]. Lastly, the increasing burden of non-communicable diseases on the continent has compelled countries to impose sin taxes to manage health care behavior among their populace [43,68]. Lobbying and advocacy from ministries of health were seen as strong support for the imposition of excise taxes. Advocacy by civil society organisations and academia was also seen as strong evidence for the imposition of such taxes as observed in South Africa [32,37].

The resistance to implementing the mechanisms could also be classified into two broad areas. First, the conflict between the healthy lives of the population and the economic growth policies [37]. Many multinational and national companies such as Coca-Cola, sugar-sweetened beverage manufacturers/importers, cigarette and cigar manufacturers/importers and beer and spirit manufacturing companies have lobbied and openly campaigned against the implementation of excise taxes that will increase their cost of import or production, thereby reducing the affordability of their goods [31,33,35]. Their argument includes creating jobs, building infrastructure and boosting the tax economy [37]. Second, resistance from the consumers was also a critical inhibitory factor. Effective intersectoral committees on non-communicable disease (NCD) excise taxes resolve such conflicts [37]. In Botswana, for example, the population that drinks alcohol had media conversations and devised strategies to oppose the excise tax on alcohol, including buying alcohol from neighboring countries and making the government look unpopular [42] in the process.

Some mechanisms are implemented as a cluster of interventions that influence health care behavior. For example, excise taxes on tobacco are implemented in tandem with smoke-free areas, age restrictions, no advertisement, and health warnings on packages [19]. Regarding alcoholic beverages, legal drinking and no drinking in public are other interventions taken to complement the excise tax. These interventions show that innovative excise tax mechanisms are not meant for fund raising alone [32,37]. Bird and Wallace [56] referred to these mechanisms as the public health model of taxing. Beyond raising funds, the model seeks to influence the consumption of alcoholic beverages, which may have adverse effects on the health and social life of the population.

### Study limitations

Data limitations did not permit us to explore the efficiency and effectiveness of the mechanisms identified. Only a few papers evaluated the use of the funds accrued from the mechanisms. It was therefore impossible to review the mechanisms based on these two criteria. The key criterion considered in this review is the additionality and allocation to the health sector. Also, we could not find data on the disbursement of funds of some existing mechanisms. They were kept on the data extraction sheet for future updates whenever data are available. Additionally, although we used multiple approaches in our search for documents, we might have missed some documents which were not in the public domain and have not been provided through expert contact. Further, it was very difficult to align findings from papers that are diverse in methodology, rigour in research, writing and publication, context, and foci. Nevertheless, to the best of our knowledge, this was the systematic effort in synthesizing innovative financing literature within the WHO Africa region.

## CONCLUSIONS

Innovative health financing mechanisms do exist and are on the increase in countries across the continent because of international policies and mechanisms that countries subscribe to because of the need to improve healthy eating and social life, and advocacy. The funds generated from many instruments are not allocated to health care, although some portions are earmarked for sports and other health-related activities. It is therefore unclear where the lines are drawn regarding what constitutes innovative health financing and to what extent funds allocated to allied health activities are considered health financing. The innovative financing mechanism that sought to impose excise taxes (sin taxes) on harmful or unhealthy products such as alcoholic beverages and sugar-sweetened drinks conflict with country-level economic growth policies and therefore receives persistent resistance from the production sector. Formulating intersectoral committees to develop and implement the innovative mechanisms involving excise taxes is highly recommended as a possible solution to the conflicts of interest.

## Additional material


Online Supplementary Document
