# Supplementary material for: The nature and contribution of innovative health financing mechanisms in the World Health Organization African region: A scoping review
Source: J Glob Health. 2023 Nov 15;13:04153. doi: 10.7189/jogh.13.04153 (PMC10644850; doi:10.7189/jogh.13.04153)
Supplement: Online Supplementary Document [file jogh-13-04153-s001.pdf]

Box S 1: Search string and link to search results

Search string: ((innovative financ*[Title/Abstract] OR sin tax[Title/Abstract] OR Tax on Insurance[Title/Abstract] OR Sugar tax[Title/Abstract] OR Salt tax[Title/Abstract] OR Mobile phone tax[Title/Abstract] OR Airline ticket tax[Title/Abstract] OR Sugar drink tax[Title/Abstract] OR Cigarrete tax[Title/Abstract]) AND (health[Title/Abstract] OR healthcare[Title/Abstract] OR health services[Title/Abstract])) AND (Africa*[Title/Abstract])

Link to search result from search string in box 1:

https://pubmed.ncbi.nlm.nih.gov/?term=%28%28innovative+financ*%5BTitle%2FAbstract%5D+OR+sin+tax%5BTitle%2FAbstract%5D+OR+Tax+on+Insurance%5BTitle%2FAbstract%5D+OR+Sugar+tax%5BTitle%2FAbstract%5D+OR+Salt+tax%5BTitle%2FAbstract%5D+OR+Mobile+phone+tax%5BTitle%2FAbstract%5D+OR+Airline+ticket+tax%5BTitle%2FAbstract%5D+OR+Sugar+drink+tax%5BTitle%2FAbstract%5D+OR+Cigarrete+tax%5BTitle%2FAbstract%5D%29+AND+%28health%5BTitle%2FAbstract%5D+OR+healthcare%5BTitle%2FAbstract%5D+OR+health+services%5BTitle%2FAbstract%5D%29%29+AND+%28Africa*%5BTitle%2FAbstract%5D%29&sort=&filter=lang.english&filter=lang.englishD%29%29+AND+%28Africa%5BTitle%2FAbstract%5D%29&filter=lang.english&size=100

Table S 2: Data matrix

No.	Author, year	Financing mechanism/ instrument	Year of inception	Setting /Country	Methodology	Facilitators	Inhibitors	Nature/ Structure	Amount (USD) raised each year 2015-2020	Lessons from development and implementation processes	Earmarked for health (Yes/No)
1.	World Health Organization [40]	Sin tax on tobacco products	Not indicated	Algeria	Report	• WHO FCTC article 6 - price and tax measures	…	34.2% excise tax on tobacco products	…	Additional tax to support emergency care	Yes
2.	World Health Organization [40]		Not indicated	Benin		• WHO FCTC article 6 - price and tax measures	…	4.9% Excise tax on tobacco		Tax did not affect smoking behaviour	
3.	World Health Organization [40]	Sin tax on Tobacco products	Not indicated	Botswana	Report	• WHO FCTC article 6 - price and tax measures	…	49.9% excise tax tobacco products	…	Not known if the tax reduced tobacco use among the population	Yes
4.	World Health Organization [40]	Sin tax on tobacco products	Not indicated	Burkina Faso	Report	• WHO FCTC article 6 - price and tax measures	…	41% excise tax on tobacco		Not known if the tax reduced tobacco use among the population	…
5.	World Health Organization [40]	Sin tax on tobacco products	Not indicated	Burundi		• WHO FCTC article 6 - price and tax measures		42.8% excise tax imposed on tobacco		Not known if the tax reduced tobacco use among	

No.	Author, year	Financing mechanism/ instrument	Year of inception	Setting /Country	Methodology	Facilitators	Inhibitors	Nature/ Structure	Amount (USD) raised each year 2015-2020	Lessons from development and implementation processes	Earmarked for health (Yes/No)
6.	World Health Organization[39] and United Nations Development Programme [46]	Sin tax on tobacco products	Not indicated	Cabo Verde	Report	• Article 6 of the WHO FCTC<br>• ECOWAS tax<br>• Ministries of health and finance worked collaboratively	• An ad valorem tax makes increasing the tax difficult	*Currently 30%–50% tax on cigarettes, cigars and waterpipe tobacco plus 20$00 escudos on each package of cigarettes*	…	There is a need to remove the ad valorem tax and make it simple for automatic increment<br><br>All the money raised is invested in sports and health	Yes
7.	Mapa-Tassou et al.[19]	Sin tax on Tobacco products	Not indicated	Cameroon		• Proposed by the health sector<br>• WHO and the establishment of the FCTC in 2003<br>• Multisectoral approach to policy formulation<br>• Political will<br>• Commitment by all stakeholders	• Lack of funds<br>• Lack of synergy among stakeholders	25% excise tax on tobacco products	…	…	No
8.	World Health Organization [40]	Sin tax on Tobacco products	Not indicated	Central African Republic	Report	• WHO and the establishment of the FCTC in 2003	•	41.5% excise tax on alcohol products	…	…	…

No.	Author, year	Financing mechanism/ instrument	Year of inception	Setting /Country	Methodology	Facilitators	Inhibitors	Nature/ Structure	Amount (USD) raised each year 2015-2020	Lessons from development and implementation processes	Earmarked for health (Yes/No)
9.	World Health Organization [40]	Sin tax on Tobacco products	Not indicated	Chad	Report	• WHO and the establishment of the FCTC in 2003	…	34.1% excise tax tobacco products	…	2% of the tax goes to antiretroviral medication	Yes
10.	World Health Organization [39]	Sin tax on tobacco products	Not indicated	Comoros	Report	• WHO and the establishment of the FCTC in 2003	…	37.3% of tobacco producer price	…	The 37.2 is lower than the 70% recommended by the WHO<br><br>All amounts raised are disbursed to Ministry of Sports and for health emergencies	Yes
11.	World Health Organization [40]	Sin tax on Tobacco products	Not indicated	Congo		WHO and the establishment of the FCTC in 2003	…	37.1% on tobacco products	…	50% of the tax is invested in health insurance	Yes
12.	World Health Organization [40]	Sin tax on Tobacco products	Not indicated	Cote d'Ivoire	Report	WHO and the establishment of the FCTC in 2003	…	33.3% excise tax on tobacco	…	Tax did not influence consumption of tobacco	…

No.	Author, year	Financing mechanism/ instrument	Year of inception	Setting /Country	Methodology	Facilitators	Inhibitors	Nature/ Structure	Amount (USD) raised each year 2015-2020	Lessons from development and implementation processes	Earmarked for health (Yes/No)
13.	World Health Organization [40]	Sin tax on Tobacco products	Not indicated	Democratic Republic of the Congo	Report	WHO and the establishment of the FCTC in 2003	…	38.7% excise tax on tobacco	…	Tax did not influence consumption of tobacco	…
14.	World Health Organization [40]	Sin tax on tobacco products	Not indicated	Equatorial Guinea	Report	WHO and the establishment of the FCTC in 2003	…	25.3% excise tax on tobacco	…	…	…
15.	World Health Organization [40]	Sin tax on tobacco products	Not indicated	Eritrea	Report	WHO and the establishment of the FCTC in 2003	…	55.4% excise tax on tobacco	…	…	…
16.	World Health Organization [40]	Sin tax on tobacco products	Not indicated	Eswatini	Report	WHO and the establishment of the FCTC in 2003	…	52.7 excise tax on tobacco	…	Tax did not influence consumption of tobacco	…
17.	Ethiopia Ministry of Health [26]; World Health Organization [40]	Sin tax on tobacco products	Instrument developed in 2018	Ethiopia	Report	• Influenced by the WHO FCTC<br>• Research evidence from the Ethiopian market<br>• Evidence shows negative influence of tobacco on health status<br>• Tax reduces consumption	There is likelihood of Ministry of Finance pushing back on the tax systems due to its impact on export revenue	To impose an extra 10% tax on cigarettes for health purposes.<br>To reduce affordability and use of tobacco products, especially among the poor	This is estimated to produce million ETB 90 per annum	The increasing price will likely influence tobacco use among the poor.<br>A strong evidence-based case to the Ministry of Finance and cabinet will be essential in	Motivate for earmarking of resources for health

No.	Author, year	Financing mechanism/ instrument	Year of inception	Setting /Country	Methodology	Facilitators	Inhibitors	Nature/ Structure	Amount (USD) raised each year 2015-2020	Lessons from development and implementation processes	Earmarked for health (Yes/No)
										implementing these taxes Specific tax better than ad valorem	
18.	World Health Organization [40]	Sin tax on tobacco products	Not indicated	Gabon	Report	• WHO and the establishment of the FCTC in 2003	…	23.1% excise tax on tobacco	…		…
19.	World Health Organization [40]	Sin tax on Tobacco products	Not indicated	Gambia	Report	• WHO and the establishment of the FCTC in 2003	…	46.3% excise tax on tobacco	…	Tax reduced the consumption of tobacco	…
20.	Parliament of Ghana [32] and Wellington [51]; World Health Organization [40]	Tobacco tax	2015	Ghana	An Act of Parliament	• WHO FCTC article 6 - price and tax measures	…	This is a 150% excise tax imposed on tobacco products (Cigarette and cigar) in 2014. This has been increased to 175% in 2015.	…	Tax did not influence tobacco use	No
21.	World Health Organization [40]; www.tobaccofreekids.org [41]	Sin tax on tobacco products	Not indicated	Equatorial Guinea	Report	• WHO FCTC article 6 - price and tax measures	…	25.3% excise tax on tobacco	…	…	…

No.	Author, year	Financing mechanism/ instrument	Year of inception	Setting /Country	Methodology	Facilitators	Inhibitors	Nature/ Structure	Amount (USD) raised each year 2015-2020	Lessons from development and implementation processes	Earmarked for health (Yes/No)
22.	World Health Organization [40]	Sin tax on tobacco products	Not indicated	Guinea-Bissau	Report	• WHO FCTC article 6 - price and tax measures		6.8% excise tax on tobacco	…	…	…
23.	World Health Organization [40]	Sin tax on tobacco products	Not indicated	Kenya	Report	• WHO FCTC article 6 - price and tax measures		52.3% excise tax on tobacco	…	Tax did not influence tobacco use	…
24.	World Health Organization [40]	Sin tax on tobacco products	Not indicated	Lesotho	Report	• WHO FCTC article 6 - price and tax measures		50.9% excise tax on tobacco	…	Tax did not influence tobacco use	…
25.	World Health Organization [40]	Sin tax on tobacco products	Not indicated	Liberia	Report	• WHO FCTC article 6 - price and tax measures		34.8% excise tax on tobacco	…	…	…
26.	World Health Organization [40]	Sin tax on tobacco products	Not indicated	Madagascar	Report	• WHO FCTC article 6 - price and tax measures	…	80.4% excise tax on tobacco	…	Tax reduced tobacco use among the population	…
27.	World Health Organization [40]; Malawi Revenue Authority [33]	Sin tax on tobacco products	2017	Malawi	Report	• WHO FCTC article 6 - price and tax measures<br>• Harmonized rate will discourage falsification of country of origin of tobacco products and encourage local		Excise tax of US$ 15 per 1000 sticks of cigarette	…	…	…

No.	Author, year	Financing mechanism/ instrument	Year of inception	Setting /Country	Methodology	Facilitators	Inhibitors	Nature/ Structure	Amount (USD) raised each year 2015-2020	Lessons from development and implementation processes	Earmarked for health (Yes/No)
						production of cigarette to produce jobs.					
28.	World Health Organization [40]	Sin tax on tobacco products	Not indicated	Mali	Report	• WHO FCTC article 6 - price and tax measures	…	27.7% excise tax on tobacco	…	Tax did not influence tobacco use	
29.	World Health Organization [40]	Tobacco tax	Not indicated	Mauritania	Report	• WHO FCTC article 6 - price and tax measures	…	9.6% excise tax on tobacco	…	Revenues from additional 7% tax on goods invested in cancer research	Yes
30.	World Health Organization [40]	Tobacco tax	Not indicated	Mauritius	Report	• WHO FCTC article 6 - price and tax measures	…	183.5% excise tax on tobacco	…	Tax reduced tobacco use	…
31.	World Health Organization [40]	Tobacco tax	Not indicated	Mozambique	Report	• WHO FCTC article 6 - price and tax measures	…	28.5% excise tax on tobacco	…	Tax reduced tobacco use	…
32.	World Health Organization [40]	Tobacco tax	Not indicated	Namibia	Report	• WHO FCTC article 6 - price and tax measures	…	44.1% excise tax on tobacco	…	…	…

No.	Author, year	Financing mechanism/ instrument	Year of inception	Setting /Country	Methodology	Facilitators	Inhibitors	Nature/ Structure	Amount (USD) raised each year 2015-2020	Lessons from development and implementation processes	Earmarked for health (Yes/No)
33.	World Health Organization [40]	Tobacco tax	Not indicated	Niger	Report	• WHO FCTC article 6 - price and tax measures	…	31.3% excise tax on tobacco	…	…	…
34.	World Health Organization [40]	Tobacco tax	Not indicated	Nigeria	Report	• WHO FCTC article 6 - price and tax measures	…	29.7%excise tax on tobacco	…	Tax did not reduce tobacco use	…
35.	World Health Organization [40]	Tobacco tax	Not indicated	Rwanda	Report	• WHO FCTC article 6 - price and tax measures	…	55.9% excise tax on tobacco	…	Tax did not reduce tobacco use	…
36.	World Health Organization [40]	Tobacco tax	Not indicated	Sao Tome and Principe	Report	• WHO FCTC article 6 - price and tax measures	…	40.4% excise tax on tobacco	…	Tax did not reduce tobacco use	…
37.	World Health Organization [40]	Tobacco tax	Not indicated	Senegal	Report	• WHO FCTC article 6 - price and tax measures	…	38.2% excise tax on tobacco	…	Tax reduced tobacco use among the population	…

No.	Author, year	Financing mechanism/ instrument	Year of inception	Setting /Country	Methodology	Facilitators	Inhibitors	Nature/ Structure	Amount (USD) raised each year 2015-2020	Lessons from development and implementation processes	Earmarked for health (Yes/No)
38.	World Health Organization [40]	Tobacco tax	Not indicated	Seychelles	Report	• WHO FCTC article 6 - price and tax measures	…	70.1% excise tax on tobacco	…	…	…
39.	World Health Organization [40]	Tobacco tax	Not indicated	Sierra Leone	Report	• WHO FCTC article 6 - price and tax measures	…	18.6% excise tax on tobacco	…	…	…
40.	Zatoński et al [20]	Tobacco tax	1993		Empirical paper (based on systematic review of media/news articles)	• Public consultation process on proposed Bill<br>• Existing legislative framework on tobacco control<br>• Evidence on burden for South Africa's economy and healthcare through tobacco-related hospitalizations, productivity loss, premature mortality and the financial impact on South African families<br>• Potential economic and health benefits	• Potential to increase illicit tobacco trade in South Africa<br>• Potential adverse impact on agriculture and township businesses<br>• Significant industry resistance and lobbying<br>• Substantial public debate	40 % excise tax plus 12 % VAT making 52% tax on tobacco in South Africa	…	• Media (print and online) plays a significant role in framing the debate on tobacco control and taxes<br>• Strong industry lobby supported by access to resources and mainstream media platforms used to drive particular	No

No.	Author, year	Financing mechanism/ instrument	Year of inception	Setting /Country	Methodology	Facilitators	Inhibitors	Nature/ Structure	Amount (USD) raised each year 2015-2020	Lessons from development and implementation processes	Earmarked for health (Yes/No)
										narrative against proposed changes<br>• Economic arguments critical of the Bill were promoted by tobacco industry spokespeople, trade unions, organizations of retailers, media celebrities and think tanks—<br>• Opportunistic and inappropriate use of data/evidence to drive anti-regulation narrative	

No.	Author, year	Financing mechanism/ instrument	Year of inception	Setting /Country	Methodology	Facilitators	Inhibitors	Nature/ Structure	Amount (USD) raised each year 2015-2020	Lessons from development and implementation processes	Earmarked for health (Yes/No)
41.	World Health Organization [40]	Tobacco tax	Not indicated	Togo	Report	• WHO FCTC article 6 - price and tax measures	• …	22.0% excise tax on tobacco	Report	•	
42.	Government of Uganda [30]; World Health Organization [40]	Excise tax on tobacco products	Act amended in 2016	Uganda	National Act	WHO FCTC article 6 - price and tax measures	…	An excise tax of 200% on tobacco and tobacco products	…	Reduced tobacco use in the population	…
43.	World Health Organization [40]	Tobacco tax	Not indicated	United Republic of Tanzania	Report	WHO FCTC article 6 - price and tax measures	…	31.2%% excise tax on tobacco	…	…	…
44.	World Health Organization [40]	Tobacco tax	Not indicated	Zambia	Report	WHO FCTC article 6 - price and tax measures	…	41.2% excise tax on tobacco	…	…	…
45.	World Health Organization [40]	Tobacco tax	Not indicated	Zimbabwe	Report	WHO FCTC article 6 - price and tax measures	…	35.9% excise tax on tobacco	…	Reduced tobacco use in the population	…
46.	World Health Organization [36] and Sornpaisarn et al.[12]	Excise tax on alcoholic beverages	Not indicated	Benin	Report			10% ad valorem tax rate on alcoholic beverages			…

No.	Author, year	Financing mechanism/ instrument	Year of inception	Setting /Country	Methodology	Facilitators	Inhibitors	Nature/ Structure	Amount (USD) raised each year 2015-2020	Lessons from development and implementation processes	Earmarked for health (Yes/No)
47.	The Global Fund [37] and Sebeelo TB [13]	Excise tax on alcoholic beverages	Not indicated	Botswana	Report	• Alcohol abuse is a key contributory factor in HIV infection<br>• Lifelong ART treatment requires sustainable funding<br>• It is least regressive<br>• Less distortionary<br>• Introduced with other regulatory mechanisms such as curfew on sales	• Increasing alcohol- related deaths<br>• Resistance from users of alcoholic beverages<br>• Increases social drinking<br>• Resulted in illegal alcohol sale and consumption<br>• Some media reported the policy was not born out of evidence	Alcoholic beverages were taxed 30% in 2008 to demotivate excessive drinking and raise funds to care for people suffering from alcohol-related ailments, and for public education on alcohol and alcohol law enforcement. The levy has increased three times to 55% by 2015.	…	Only 10% of the funds go to the Ministry of Health.<br>45% goes to the consolidated fund and 45% to the Ministry of Youth.	Yes
48.	World Health Organization [36]; Bird & Wallace [38]	Excise tax on alcoholic beverages	Not indicated	Burkina Faso	Report	…	…	Excise tax of 25% on beer, wine and distilled spirits	…	…	…
49.	World Health Organization [39] Pricewaterhouse Coopers [44]	Excise tax on alcoholic beverages	Not indicated	Cabo Verde	Report	…	…	*A 40% excise tax on alcoholic beverages*	…	…	No

No.	Author, year	Financing mechanism/ instrument	Year of inception	Setting /Country	Methodology	Facilitators	Inhibitors	Nature/ Structure	Amount (USD) raised each year 2015-2020	Lessons from development and implementation processes	Earmarked for health (Yes/No)
50.	World Health Organization [36]	Excise tax on alcoholic beverages	Not indicated	Cameroon	Report	…	…	…	…	…	…
51.	World Health Organization [36]	Excise tax on alcoholic beverages	Not indicated	Central African Republic	Report	…	…	…	…	…	…
52.	World Health Organization [36]	Excise tax on alcoholic beverages	Not indicated	Chad	Report	…	…	…	…	…	…
53.	World Health Organization[39]	Excise tax on alcoholic beverages	Not indicated	Comoros	Report	…	…	…	…	…	…
54.	World Health Organization [36]	Excise tax on alcoholic beverages	Not indicated	Congo	Report	…	…	…	…	…	…
55.	World Health Organization [36]	Excise tax on alcoholic beverages	Not indicated	Democratic Republic of the Congo	Report	…	…	…	…	…	…
56.	World Health Organization [36]	Excise tax on alcoholic beverages	Not indicated	Côte d’Ivoire	Report	…	…	…	…	…	…

No.	Author, year	Financing mechanism/ instrument	Year of inception	Setting /Country	Methodology	Facilitators	Inhibitors	Nature/ Structure	Amount (USD) raised each year 2015-2020	Lessons from development and implementation processes	Earmarked for health (Yes/No)
57.	World Health Organization [36]	Excise tax on alcoholic beverages	Not indicated	Equatorial Guinea	Report	…	…	…	…	…	…
58.	World Health Organization [36]	Excise tax on alcoholic beverages	Not indicated	Eretria	Report	…	…	…	…	…	…
59.	World Health Organization [36]	Excise tax on alcoholic beverages	Not indicated	Eswatini	Report	…	…	…	…	…	…
60.	World Health Organization [36] and Ethiopia Ministry of Health [26]	Imposing 5% increase on alcoholic beverages	Developed in 2018	Ethiopia	Report	• Potential improvement in the health of population.<br>Ministry of health led	…	An additional excise tax	1.8 billion per annum	A strong evidence-based case to the Ministry of Finance and cabinet will be essential in implementing these taxes	Urge earmarking of resources for health
61.	World Health Organization [36]	Excise tax on alcoholic beverages	Not indicated	Gabon	Report	…	…	…	…	…	…
62.	World Health Organization [36]	Excise tax on alcoholic beverages	Not indicated	Gambia	Report	…	…	…	…	…	…

No.	Author, year	Financing mechanism/ instrument	Year of inception	Setting /Country	Methodology	Facilitators	Inhibitors	Nature/ Structure	Amount (USD) raised each year 2015-2020	Lessons from development and implementation processes	Earmarked for health (Yes/No)
63.	World Health Organization [36] and Bird & Wallace [38]	Excise tax on alcoholic beverages	Not indicated	Ghana	Report	…	…	30% on beer and 25% on spirits	…	…	
64.	World Health Organization [36]	Excise tax on alcoholic beverages	Not indicated	Guinea	Report	…	…	Imposed 5% on beer and 30% on distilled spirits	…	…	…
65.	National Council for Law Reporting [27]	Excise tax on alcoholic beverages	2015	Kenya	National Act	Negative effects of alcohol on the Kenyan population	Resistance from the alcohol industry.	Imposed KSh 150 on wines and fortified wines and other fermented fruits and KSh 200 per litre on spirits and other alcoholic beverages with more than 10% alcohol content.<br><br>This price is reviewed annually.		Kenya has recently imposed more tax on alcohol advertisement with the purpose of curbing excessive alcohol consumption	
66.	World Health Organization [36] and Bird & Wallace [38]	Excise tax on alcoholic beverages	Not indicated	Lesotho	Report	…	…	Imposed 2.239 cents per litre of beer; 169 cents per litre of fortified wine, 77 cents per litre of	…	…	…

No.	Author, year	Financing mechanism/ instrument	Year of inception	Setting /Country	Methodology	Facilitators	Inhibitors	Nature/ Structure	Amount (USD) raised each year 2015-2020	Lessons from development and implementation processes	Earmarked for health (Yes/No)
								imported unfortified wine and 7.82 cents per litre of imported unfortified wine; and 254.8 cents per litre of spirits.			
67.	World Health Organization [36]	Excise tax on alcoholic beverages	Not indicated	Liberia	Report	…	…	…	…	…	…
68.	World Health Organization [45]	Excise tax on alcoholic beverages		Madagascar	Policy brief	…	…	…	…		
69.	World Health Organization [36] and Bird & Wallace [38]	Excise tax on alcoholic beverages	Not indicated	Malawi	Report	…	…	Imposed 15% excise tax on opaque beer and 30% on others; 65% on wines; and 65% on spirits	…	…	…
70.	World Health Organization [36] and Bird & Wallace [38]	Excise tax on alcoholic beverages	Not indicated	Mali	Report	…	…	Imposed 5% on all alcoholic beverages	…	…	…

No.	Author, year	Financing mechanism/ instrument	Year of inception	Setting /Country	Methodology	Facilitators	Inhibitors	Nature/ Structure	Amount (USD) raised each year 2015-2020	Lessons from development and implementati on processes	Earmarked for health (Yes/No)
71.	World Health Organization [36]	Excise tax on alcoholic beverages	Not indicated	Mauritius	Report	…	…	An excise tax of 40.3% of retail process of beer, 11.9% of retail price of wine and 62.5% of retail price of spirits	…	…	…
72.	World Health Organization [36]	Excise tax on alcoholic beverages	Not indicated	Namibia	Report	…	…	…	…	…	…
73.	World Health Organization [36] and Bird & Wallace [38]	Excise tax on alcoholic beverages	Not indicated	Niger	Report	…	…	Imposed 25% excise tax on malt beer; 45% on other forms of beer; wine and Spirits.	…	…	…
74.	World Health Organization [36]	Excise tax on alcoholic beverages	Not indicated	Nigeria	Report	…	…	…	…	…	…
75.	World Health Organization [36]	Excise tax on alcoholic beverages	Not indicated	Rwanda	Report	…	…	Imposed 57% on domestically produced beer, 220% on imported Amstel, CIF); and 57% on other imported beer; 70% on	…	…	…

No.	Author, year	Financing mechanism/ instrument	Year of inception	Setting /Country	Methodology	Facilitators	Inhibitors	Nature/ Structure	Amount (USD) raised each year 2015-2020	Lessons from development and implementation processes	Earmarked for health (Yes/No)
								wine and 30% on spirits.			
76.	World Health Organization [36]	Excise tax on alcoholic beverages	Not indicated	Sao Tome and Principle	Report	…	…	…	…	…	…
77.	Alcohol and tobacco tax and trade bureau 2011 [28]	Excise tax on alcoholic beverages		Senegal	Website	…	…	Imposed 20% tariff with 1% statistical tax, 1% community solidarity levy and 18% VAT on alcohol products. High concentration of alcohol also attracts extra tax	…	…	…
78.	World Health Organization [36]	Excise tax on alcoholic beverages	Not indicated	Seychelles	Report	…	…	…	…	…	…
79.	World Health Organization [36]	Excise tax on alcoholic beverages	Not indicated	Sierra Leone	Report	…	…	…	…	…	…
80.	World Health Organization [36]	Excise tax on alcoholic beverages	Not indicated	South Africa	Report (web-based dataset)	• Positive health benefits<br>• Positive spillover effects (reductions in GBV, violence, MVAs, etc.)	• Strong lobby from alcohol industry<br>• Downstream effects on suppliers	R 46.4 per litre of malt/clear beer and 7.8 cent per litre of traditional beer, R3.7 per litre of fortified wine, R6.2 per litre of	Not indicated	In addition to appropriate excise taxation policies, there is a need for complementary non-tax	No

No.	Author, year	Financing mechanism/ instrument	Year of inception	Setting /Country	Methodology	Facilitators	Inhibitors	Nature/ Structure	Amount (USD) raised each year 2015-2020	Lessons from development and implementation processes	Earmarked for health (Yes/No)
							(increase in costs, etc.)<br>• Upstream effects on township businesses (price increases could lead to reduced demand)	sparkling wine, R2.0 on unfortified wine, and R 77.7 on spirits		measures that can be effectively targeted at specific consumers and high-risk behaviour patterns.<br><br>Educational programmes and regulatory interventions aimed at discouraging risky and hazardous alcohol consumption are necessary to complement the alcohol excise tax regime.	
81.	United Republic of Tanzania [29]	Excise tax on alcoholic beverages	Not indicated	United Republic of Tanzania	Government Gazette	…	…	Ad valorem of TSh 540 per litre on spirits distilled locally from grapes;	…	…	…

No.	Author, year	Financing mechanism/ instrument	Year of inception	Setting /Country	Methodology	Facilitators	Inhibitors	Nature/ Structure	Amount (USD) raised each year 2015-2020	Lessons from development and implementation processes	Earmarked for health (Yes/No)
								TSh 3978 on other locally produced spirits; TSh 4386.06 on spirits from distilling grape wine; TSh 2386 per litre on whiskies; 4386.06 per litre on spirits distilled from sugar-cane; TSh 4386 per litre of Gin, vodka, liqueurs, cordials and other spirits.			
82.	World Health Organization [36]; Bird & Wallace [5]	Excise tax on alcoholic beverages	Not indicated	Togo	Report	…	…	Imposed 10% excise tax on beer, 15% on wine and spirits	…	…	…
83.	World Health Organization [36]; Government of Uganda [30]; Bird & Wallace [5]	Excise tax on alcoholic beverages	Not indicated	Uganda	Report	…	…	An excise tax of 60% on beer (malt; 30% on beer using 75% or above of local materials; 60 % on imported beer;	…	…	…

No.	Author, year	Financing mechanism/ instrument	Year of inception	Setting /Country	Methodology	Facilitators	Inhibitors	Nature/ Structure	Amount (USD) raised each year 2015-2020	Lessons from development and implementation processes	Earmarked for health (Yes/No)
								60% on wine and Spirits.			
84.	World Health Organization [36] and Mukanu et al [18]	Excise tax on alcoholic beverages	Not indicated	Zambia		• The industry understood the need to tax beverages	• The sin tax is in opposition to the economic policy of the country	A 35%-75% excise tax imposed on alcoholic beverages was reduced to 60% in 2009 and to 40% in 2011.			…
85.	Zimbabwean Government 2021 [31]		Not indicated	Zimbabwe		Sales of alcohol massively increased from 2019 to 2020. Political will and the need to curb NCDs.	…	30% retail price plus ZW$ 10.00/L	…	The Vice-President, who is also the Minister of Health proposed increasing the excise tax to fund health. The tax system should change from type of alcohol to tax according to the alcoholic content of the drink.	

No.	Author, year	Financing mechanism/ instrument	Year of inception	Setting /Country	Methodology	Facilitators	Inhibitors	Nature/ Structure	Amount (USD) raised each year 2015-2020	Lessons from development and implementation processes	Earmarked for health (Yes/No)
86.	The Global Fund [37] and Carter Center [42]	Airline levies: US$ 1–US$ 2 on economy and US$ 10 on business class air tickets. Initiated by the country and coordinated by UNITAID to fund healthcare	After 2006	Benin	Report	• It was scientifically proven that it has no influence on air traffic<br>• Legislative review and clarity<br>• It is a transparent way of generating money<br>• Cost effective to generate<br>• Acceptable to contributors	• High-level political commitment<br>• Good leadership<br>• Evidence-based<br>• Poor stakeholder consultation<br>• Slow implementation	• Country makes the policy.<br>• UNITAID coordinates<br>• Airlines collect and declare tax<br>• Funds collected from the airlines are transferred to the national treasury for use	…	This levy is used to fight HIV/AIDS and tuberculosis.<br><br>Other African countries considering a similar mechanism. They include Chad, Kenya, Mozambique and Nigeria	Yes
87.	The Global Fund [37] and Carter [42]	Airline levies: US$ 1–US$ 2 on economy and US$ 10 on business class air tickets. Initiated by the country and coordinated by UNITAID to fund healthcare	After 2006	Burkina Faso	Report	• It was scientifically proven that it has no influence on air traffic<br>• Legislative review and clarity<br>• It is a transparent way of generating money<br>• Cost effective to generate<br>• Acceptable to contributors	• High-level political commitment<br>• Good leadership<br>• Evidence based	• Country makes the policy<br>• UNITAID coordinates<br>• Airlines collect and declare tax<br>• Funds collected from the airlines are transferred to the national treasury for use	…	This levy is used to fight HIV/AIDS and tuberculosis programmes.	Yes

No.	Author, year	Financing mechanism/ instrument	Year of inception	Setting /Country	Methodology	Facilitators	Inhibitors	Nature/ Structure	Amount (USD) raised each year 2015-2020	Lessons from development and implementation processes	Earmarked for health (Yes/No)
88.	The Global Fund [37] and Carter [42]	Airline levies: US$ 1–US$ 2 on economy and US$ 10 on business class air tickets. Initiated by the country and coordinated by UNITAID to fund healthcare	2006	Cameroon	Report	• It was scientifically proven that it has no influence on air traffic<br>• Legislative review and clarity<br>• It is a transparent way of generating money<br>• Cost effective to generate<br>• Acceptable to contributors	• High-level political commitment<br>• Good leadership<br>• Evidence-based	• Country makes the policy.<br>• UNITAID coordinates<br>• Airlines collect and declare tax<br>• Funds collected from the airlines are transferred to the national treasury for use	**2006-2012** Cameroon: 1 430 000<br><br>2011:2018	…	Yes
89.	The Global Fund [37] and Carter Center [42]	Airline levies: $1–$2 on economy and US$ 10 on business class air tickets. Initiated by country and coordinated by UNITAID to fund healthcare	After 2006	Central African Republic	Report	• It was scientifically proven that it has no influence on air traffic<br>• Legislative review and clarity<br>• It is a transparent way of generating money<br>• Cost effective to generate<br>• Acceptable to Contributors	• High-level political commitment<br>• Good leadership<br>• Evidence-based	• Country makes the policy<br>• UNITAID coordinates<br>• Airlines collect and declare tax<br>• Funds collected from the airlines are transferred to the national treasury for use	…	…	Yes

No.	Author, year	Financing mechanism/ instrument	Year of inception	Setting /Country	Methodology	Facilitators	Inhibitors	Nature/ Structure	Amount (USD) raised each year 2015-2020	Lessons from development and implementati on processes	Earmarked for health (Yes/No)
90.	The Global Fund [37] and Carter Center [42]	Airline levies: $1–$2 on the economy and US$ 10 on business class air tickets. Initiated by the country and coordinated by UNITAID to fund healthcare	2006	Democratic Republic of the Congo	Report	• It was scientifically proven that it has no influence on air traffic<br>• Legislative review and clarity<br>• It is a transparent way of generating money<br>• Cost effective to generate<br>• Acceptable to contributors	• High-level political commitment<br>• Good leadership<br>• Evidence-based	• Country makes the policy.<br>• UNITAID coordinates<br>• Airlines collect and declare tax<br>• Funds collected from the airlines are transferred to the national treasury for use	**2006-2012** Congo: 1 090 000	This levy is used to fight HIV/AIDS and tuberculosis programmes.	Yes
91.	The Global Fund [37] and Carter Center [42]	Airline levies: US$ 1–US$ 2 on economy and US$ 10 on business class air tickets. Initiated by country and coordinated by UNITAID to fund healthcare	After 2006	Côte d'Ivoire	Report	• It was scientifically proven that it has no influence on air traffic<br>• Legislative review and clarity<br>• It is a transparent way of generating money<br>• Cost effective to generate<br>• Acceptable to contributors	• High-level political commitment<br>• Good leadership<br>• Evidence based	• Country makes the policy.<br>• UNITAID coordinates<br>• Airlines collect and declare tax<br>• Funds collected from the airlines are transferred to the national treasury for use	…	…	Yes

No.	Author, year	Financing mechanism/ instrument	Year of inception	Setting /Country	Methodology	Facilitators	Inhibitors	Nature/ Structure	Amount (USD) raised each year 2015-2020	Lessons from development and implementation processes	Earmarked for health (Yes/No)
92.	The Global Fund [37] and Carter Center [42]	Airline levies: US$ 1–US$ 2 on economy and US$ 10 on business class air tickets. Initiated by the country and coordinated by UNITAID to fund healthcare	After 2006	Gabon	Report	• It was scientifically proven that it has no influence on air traffic<br>• Legislative review and clarity<br>• It is a transparent way of generating money<br>• Cost effective to generate<br>• Acceptable to contributors	• High level political commitment<br>• Good leadership<br>• Evidence-based	• Country makes the policy.<br>• UNITAID coordinates<br>• Airlines collect and declare tax<br>• Funds collected from the airlines are transferred to the national treasury for use	…	…	Yes
93.	The Global Fund[37] and Carter Center [42]	Airline levies: $1–$2 on economy and US$ 10 on business class air tickets. Initiated by the country and coordinated by UNITAID to fund healthcare	2006	Guinea	Report	• It was scientifically proven that it has no influence on air traffic<br>• Legislative review and clarity<br>• It is a transparent way of generating money<br>• Cost effective to generate<br>• Acceptable to contributors	• High-level political commitment<br>• Good leadership<br>• Evidence based	• Country makes the policy.<br>• UNITAID coordinates<br>• Airlines collect and declare tax<br>• Funds collected from the airlines are transferred to the national treasury for use	**2006-2012** Guinea: 49 000	…	Yes

No.	Author, year	Financing mechanism/ instrument	Year of inception	Setting /Country	Methodology	Facilitators	Inhibitors	Nature/ Structure	Amount (USD) raised each year 2015-2020	Lessons from development and implementation processes	Earmarked for health (Yes/No)
94.	The Global Fund [37] and Carter Center [42]	Airline levies: US$ 1–US$ 2 on the economy and US$ 10 on business class air tickets. Initiated by the country and coordinated by UNITAID to fund healthcare	After 2006	Liberia	Report	• It was scientifically proven that it has no influence on air traffic<br>• Legislative review and clarity<br>• It is a transparent way of generating money<br>• Cost effective to generate<br>• Acceptable to Contributors	• High-level political commitment<br>• Good leadership<br>• Evidence based	• Country makes the policy<br>• UNITAID coordinates<br>• Airlines collect and declare tax<br>• Funds collected from the airlines are transferred to the national treasury for use	…	…	Yes
95.	The Global Fund [37] and Carter Center [42]	Airline Levies: US$ 1–US$ 2 on economy and US$ 10 on business class air tickets. Initiated by the country and coordinated by UNITAID to fund healthcare	2006	Madagascar	Report	• It was scientifically proven that it has no influence on air traffic<br>• Legislative review and clarity<br>• It is a transparent way of generating money<br>• Cost effective to generate<br>• Acceptable to contributors	• High-level political commitment<br>• Good leadership<br>• Evidence-based	• Country makes the policy<br>• UNITAID coordinates<br>• Airlines collect and declare tax<br>• Funds collected from the airlines are transferred to the national treasury for use	**2006-2012** Madagascar : 30 000	…	Yes

No.	Author, year	Financing mechanism/ instrument	Year of inception	Setting /Country	Methodology	Facilitators	Inhibitors	Nature/ Structure	Amount (USD) raised each year 2015-2020	Lessons from development and implementation processes	Earmarked for health (Yes/No)
96.	The Global Fund[37] and Carter Center [42]	Airline Levies: US$ 1–US$ 2 on economy and US$ 10 on business class air tickets. Initiated by the country and coordinated by UNITAID to fund healthcare	2006	Mali	Report	• It was scientifically proven that it has no influence on air traffic<br>• Legislative review and clarity<br>• It is a transparent way of generating money<br>• Cost effective to generate<br>• Acceptable to contributors	• High-level political commitment<br>• Good leadership<br>• Evidence based	• Country makes the policy.<br>• UNITAID coordinates<br>• Airlines collect and declare tax<br>• Funds collected from the airlines are transferred to the national treasury for use	**2006-2012** Mali: 928 000	…	Yes
97.	The Global Fund[37] and Carter Center [42]	Airline Levies: US$ 1–US$ 2 on economy and US$ 10 on business class air tickets. Initiated by the country and coordinated by UNITAID to fund healthcare	2006	Mauritius	Report	• It was scientifically proven that it has no influence on air traffic<br>• Legislative review and clarity<br>• It is a transparent way of generating money<br>• Cost effective to generate<br>• Acceptable to contributors	• High-level political commitment<br>• Good leadership<br>• Evidence based	• Country makes the policy.<br>• UNITAID coordinates<br>• Airlines collect and declare tax<br>• Funds collected from the airlines are transferred to the national treasury for use	**2006–2012** q: 8 741 000	…	Yes

No.	Author, year	Financing mechanism/ instrument	Year of inception	Setting /Country	Methodology	Facilitators	Inhibitors	Nature/ Structure	Amount (USD) raised each year 2015-2020	Lessons from development and implementation processes	Earmarked for health (Yes/No)
98.	The Global Fund[37] and Carter Center [42]	Airline levies: US$ 1–US$ 2 on economy and US$ 10 on business class air tickets. Initiated by the country and coordinated by UNITAID to fund healthcare	After 2006	Namibia	Report	• It was scientifically proven that it has no influence on air traffic<br>• Legislative review and clarity<br>• It is a transparent way of generating money<br>• Cost effective to generate<br>• Acceptable to contributors	• High-level political commitment<br>• Good leadership<br>• Evidence-based	• Country makes the policy.<br>• UNITAID coordinates<br>• Airlines collect and declare tax<br>• Funds collected from the airlines are transferred to the national treasury for use		…	Yes

No.	Author, year	Financing mechanism/ instrument	Year of inception	Setting /Country	Methodology	Facilitators	Inhibitors	Nature/ Structure	Amount (USD) raised each year 2015-2020	Lessons from development and implementation processes	Earmarked for health (Yes/No)
99.	The Global Fund[37] and Carter Center [42]	Airline Levies: US$ 1–US$ 2 on Economic and 10 USD on business class air tickets. Initiated by the country and coordinated by UNITAID to fund healthcare	2006	Niger	Report	• It was scientifically proven that it has no influence on air traffic<br>• Legislative review and clarity<br>• It is a transparent way of generating money<br>• Cost effective to generate<br>• Acceptable to Contributors	• High-level political commitment<br>• Good leadership<br>• Evidence based	• Country makes the policy.<br>• UNITAID coordinates<br>• Airlines collect and declare tax<br>• Funds collected from the airlines are transferred to the national treasury for use	**2006-2012** Niger: 281 000	…	Yes

No.	Author, year	Financing mechanism/ instrument	Year of inception	Setting /Country	Methodology	Facilitators	Inhibitors	Nature/ Structure	Amount (USD) raised each year 2015-2020	Lessons from development and implementation processes	Earmarked for health (Yes/No)
100.	The Global Fund [37] and Carter Center [42]	• Mandatory solidarity levy on airline tickets<br>• Airline levies: US$ 1–US$ 2 on economy and US$ 10 on business class air tickets.<br>• Initiated at country level and coordinated by UNITAID to fund healthcare	After 2006	South Africa	Report	• It was scientifically proven that it has no influence on air traffic<br>• Legislative review and clarity<br>• It is a transparent way of generating money<br>• Cost effective to generate<br>• Acceptable to contributors - the levy is a relatively small amount	• High-level political commitment<br>• Good leadership<br>• Evidence based	• Country makes the policy<br>• UNITAID coordinates<br>• Airlines collect and declare tax<br>• Funds collected from the airlines are transferred to the national treasury for use	Not reported *(figures in report are for the period 2006 -2012)*	• Economically neutral, equitable and transparent.<br>• Research has shown that it has no negative effect on air traffic, airline jobs or profitability.<br>• Administratively simple and cost effective.<br>• Positive reception among passengers and tax payers	Yes<br>*(UNITAID funds programmes for AIDS, tuberculosis and malaria in developing countries.)*

No.	Author, year	Financing mechanism/ instrument	Year of inception	Setting /Country	Methodology	Facilitators	Inhibitors	Nature/ Structure	Amount (USD) raised each year 2015-2020	Lessons from development and implementation processes	Earmarked for health (Yes/No)
101.	The Global Fund [37] and Carter Center [42]	Airline levies: US$ 1–US$ 2 on economy and US$ 10 on business class air tickets.<br><br>Initiated at country level and coordinated by UNITAID to fund healthcare	After 2006	Sao Tome and Principe	Report	• It was scientifically proven that it has no influence on air traffic<br>• Legislative review and clarity<br>• It is a transparent way of generating money<br>• Cost effective to generate<br>• Acceptable to contributors	• High-level political commitment<br>• Good leadership<br>• Evidence based	• Country makes the policy<br>• UNITAID coordinates<br>• Airlines collect and declare tax<br>• Funds collected from the airlines are transferred to the national treasury for use	…	…	Yes
102.	The Global Fund [37] and Carter Center [42]	Airline levies: US$ 1–US$ 2 on economy and US$ 10 on business class air tickets Initiated by country and coordinated by UNITAID to fund healthcare	After 2006	Senegal	Report	• It was scientifically proven that it has no influence on air traffic<br>• Legislative review and clarity<br>• It is a transparent way of generating money<br>• Cost effective to generate<br>• Acceptable to contributors	• High-level political commitment<br>• Good leadership<br>• Evidence based	• Country makes the policy<br>• UNITAID coordinates<br>• Airlines collect and declare tax<br>• Funds collected from the airlines are transferred to the national treasury for use	…	…	Yes

No.	Author, year	Financing mechanism/ instrument	Year of inception	Setting /Country	Methodology	Facilitators	Inhibitors	Nature/ Structure	Amount (USD) raised each year 2015-2020	Lessons from development and implementation processes	Earmarked for health (Yes/No)
103.	The Global Fund [37] and Carter Center [42]	Airline levies: US$ 1–US$ 2 on economy and US$ 10 on business class air tickets. Initiated at country level and coordinated by UNITAID to fund healthcare	After 2006	Togo	Report	• It was scientifically proven that it has no influence on air traffic<br>• Legislative review and clarity<br>• It is a transparent way of generating money<br>• Cost effective to generate<br>• Acceptable to contributors	• High-level political commitment<br>• Good leadership<br>• Evidence-based	• Country makes the policy<br>• UNITAID coordinates<br>• Airlines collect and declare tax<br>• Funds collected from the airlines are transferred to the national treasury for use	…	…	Yes
104.	Thow et. al. [21]	Excise tax of KSh 20 ($0.20) on sugar confectionery	…	Kenya	• Policy analysis	• Growing rate of noncommunicable diseases<br>• Food security<br>• International policies	• Economic policies	Excise tax of KSh 20 ($ 0.20) on sugar confectionery	…	…	…
105.	Beebeejaun [22]	Sugar-based beverage taxes<br><br>3 cents per gram of sugar in sugar-sweetened drinks both imported or locally	2016	Mauritius	Empirical study	• Noncommunicable disease burden<br>• Government commitment	…	Specific excise tax/duty Currently 6 cents per gram of sugar. Drinks being exported are exempted from the tax. This is to reduce the consumption of	…	The levy resulted in reduction in sugar consumption. Recommendations were made for similar tax on salt and fatty foods.	No

No.	Author, year	Financing mechanism/ instrument	Year of inception	Setting /Country	Methodology	Facilitators	Inhibitors	Nature/ Structure	Amount (USD) raised each year 2015-2020	Lessons from development and implementation processes	Earmarked for health (Yes/No)
		manufactured from 2016.						unhealthy food, and in effect, lower the risk of noncommunicable diseases in Mauritius.			
106.	. Federal Republic of Nigeria [34]	Excise tax on sugar-sweetened beverages (SSBs)	2021	Nigeria	• Government gazette	…	…	An excise tax of N10 per litre on all carbonated and sugar-sweetened drinks	…	…	…
107.	.	Excise tax of 39% on soft drinks, including non-SSBs	…	Rwanda	• Policy analysis	• Growing rate of noncommunicable diseases<br>• International policies	• Economic policies	Excise tax of 39% on soft drinks, including non-SSBs, with revenue earmarked broadly for Government	…	Earmarked broadly for Government	Yes
108.	Republic of South Africa [35]; Stacey et al. [23]; Stacey et al. [24] & Karim, Kruger & Hofman [25]	Sugar-based beverage taxes implemented in 2018. This constitutes $0.15 per gram of sugar in sugar-sweetened beverages. This	2017	South Africa	Empirical papers	• Positive impact on healthy living<br>• Easily implementable<br>• Transparent system	• Opposition from the sugar industry<br>• Affects the income of sugar growers<br>• Some job losses	The first 4g per every 100 ml is not charged. Subsequent grammes are charged per the prevailing rate (2.21 SA cents) per gram.	First year: R2 billion	• Requires a protracted legislative process, including extensive public consultations<br>•	No<br><br>(although some of the revenue raised from the HPL will be “soft-earmarked”

No.	Author, year	Financing mechanism/ instrument	Year of inception	Setting /Country	Methodology	Facilitators	Inhibitors	Nature/ Structure	Amount (USD) raised each year 2015-2020	Lessons from development and implementation processes	Earmarked for health (Yes/No)
		was done under the Health Promotion Levy (HPL) to reduce the high intake of sugar-sweetened beverages with their associated health risks.					• Increases the cost of beverages by 11%<br>• Price of sugar cane reduced	The tax is charged at the source of manufacture<br><br>Money generated is transferred to national revenue fund.<br><br>The rate is adjusted yearly to cater for inflation		• Households in urban communities reduced their sugar-sweetened drinks consumption by half.<br>• Compared to nontaxable beverages, consumer purchases of taxable beverages reduced significantly after the implementation of the policy.<br>• The threshold approach is an incentive for manufacturer	for health promotion activities across government.

No.	Author, year	Financing mechanism/ instrument	Year of inception	Setting /Country	Methodology	Facilitators	Inhibitors	Nature/ Structure	Amount (USD) raised each year 2015-2020	Lessons from development and implementations on processes	Earmarked for health (Yes/No)
										s to shift to lower sugar content beverages to avoid tax.	
109.	102. Thow et al.	Excise tax on nonalcoholic beverages and levy on imports	…	United Republic of Tanzania	• Policy analysis	• Growing rate of noncommunicable diseases<br>• International policies	• Economic policies	TSh 54 ($0.02) per litre on nonalcoholic beverages and levy on imports	…	Earmarked broadly for Government	Yes
110.	103. Thow et al. [21]	Excise tax on nonalcoholic beverages and levy on sugar	…	Uganda	• Policy analysis; Government Gazette	• Growing rate of noncommunicable diseases<br>• International policies	• Economic policies	60% excise tax on alcohol and 80% on spirits	…	Earmarked for HIV/AIDS Trust Fund	Yes
111.	104. Thow et al. [14]; Thow et al.[21]; Hangoma et al [15] ; Mukanu et al [18]<br>105.	Sugar tax in Zambia in 1998.	…	Zambia		Strong lobbying from the health sector Opportunities exist to strengthen the policy through broad-based consultation	• The general public was poorly educated about the tax<br>• The policy conflicts with economic growth policies<br>• Pushback from Government economic policies sector and industry	*25% excise tax on sugar-sweetened beverages was introduced in 1998. This was repealed in 2015 to favour economic policies. Currently 3% on imported beverages and 0.5% locally manufactured drinks*	…	The studies and economic modelling predicted 25% excise tax on SSBs, which will have a positive impact on the consumption of SSBs in Zambia but the final policy only imposes $0.02) (3%) excise tax on	…

No.	Author, year	Financing mechanism/ instrument	Year of inception	Setting /Country	Methodology	Facilitators	Inhibitors	Nature/ Structure	Amount (USD) raised each year 2015-2020	Lessons from development and implementation processes	Earmarked for health (Yes/No)
										SSBs. Compared to the 20% recommended by WHO, this is way too low.	
112.	106. Carter Center [42]	Micro levy on oil, gas, gold and other mining activities	2015	Republic of the Congo	Report	• Acceptable to contributors<br>• It is a transparent way of generating money<br>• Cost effective to generate	• Lack of result management<br>• Uneven application of value for money criteria	UNITLIFE introduced 0.1% levies on oil, gas, gold and other mining activities to raise funds for nutrition.<br><br>*The Democratic Republic of the Cong imposed 0.10 USD per barrel of oil sold.*<br>*This has the potential to raise* USD100-200 million annually in Africa	…	…	Yes

No.	Author, year	Financing mechanism/ instrument	Year of inception	Setting /Country	Methodology	Facilitators	Inhibitors	Nature/ Structure	Amount (USD) raised each year 2015-2020	Lessons from development and implementati on processes	Earmarked for health (Yes/No)
113.	. Carter Center [42]	Micro levy on oil, gas, gold and other mining activities	2015	Guinea	Report	• Acceptable to contributors<br>• It is a transparent way of generating money<br>• Cost effective to generate	• Lack of result management<br>• Uneven application of value for money criteria	UNITLIFE introduced 0.1% levies on oil, gas, gold and other mining activities to raise funds for nutrition.	…	…	Yes
114.	. Carter Center	Micro levy on oil, gas, gold and other mining activities	2015	Mali	Report	• Acceptable to contributors<br>• It is a transparent way of generating money<br>• Cost effective to generate	• Lack of result management<br>• Uneven application of value for money criteria	UNITLIFE introduced 0.1% levies on oil, gas, gold and other mining activities to raise funds for nutrition.	…	…	Yes
115.	. Carter Center [42]	Micro levy on oil, gas, gold and other mining activities	2015	Niger	Report	• Acceptable to contributors<br>• It is a transparent way of generating money<br>• Cost effective to generate	• Lack of result management<br>• Uneven application of value for money criteria	UNITLIFE introduced 0.1% levies on oil, gas, gold and other mining activities to raise funds for nutrition.	…	…	Yes
116.	Atun et al. [16] and The Global Fund [37]	AIDS Trust Fund (a tax/levy-based instrument)	2000	Zimbabwe	A systematic review to identify innovative financing in SSA	• Advocacy by people living with HIV<br>• Established as a seed fund to attract donor funding	• Strict financial plans that allocate majority of funds to ARVs limit expenditure	Trust fund 3% levy on PAYE and 3% on income tax for corporate bodies established by	2012: 32 640 678<br>2013: 34 236 005<br>2014: 35,5 million	• Levy is deposited into NAC's account directly	Yes

No.	Author, year	Financing mechanism/ instrument	Year of inception	Setting /Country	Methodology	Facilitators	Inhibitors	Nature/ Structure	Amount (USD) raised each year 2015-2020	Lessons from development and implementation processes	Earmarked for health (Yes/No)
						• Strong cooperative governance<br>• Clearly defined use of funds<br>• Annual workplan and budget<br>• Fund administered according to laid down policies<br>• Fund decentralized to the lowest level<br>• Active participation of district AIDS action committees	on health promotion<br>• 70% of the population employed in the informal sector are not taxed.<br>• Funds are affected by hyperinflation	National AIDS Council (NAC) of Zimbabwe Act, to tackle the burden of HIV/AIDs in Zimbabwe	This accounted for 15% of the health expenditure on HIV/AIDS.	• Advocacy by the population in need plays a key role in sourcing funds.<br>• Countries making sustainable commitment to healthcare have a potential for attracting donor funding.<br>• Funds have been used transparently and solely for the purpose for which they were raised.<br>• This is considered a best practice in Africa. It provided the	

No.	Author, year	Financing mechanism/ instrument	Year of inception	Setting /Country	Methodology	Facilitators	Inhibitors	Nature/ Structure	Amount (USD) raised each year 2015-2020	Lessons from development and implementation processes	Earmarked for health (Yes/No)
										NAC resilience through economic recession.<br>• There was accountability through the media updates, generating trust in the scheme	
117.	110. Birungi & Colbourn [66] and Koseki, Fagan & Menonc [47]	**HIV/AIDS Trust Fund**	2012	Uganda	Policy document	• Strong political will	• Delay in implementation due to bureaucracy<br>• Lack of clarity in policy<br>• Funds may not be used for HIV if clearly defined roles are not imputed<br>• Only 0.5% of HIV expenditure is covered<br>• There was insufficient	***Trust Fund*** established by an Act of parliament- The HIV and AIDS Prevention and Control Act<br><br>2% tax on alcohol, soft drinks, beer and bottled water to fund HIV care	Projected $2 million per annum which translates into 0.5% of HIV expenditure.	Although the policy provides a sustainable source of funding, there are questions regarding stakeholder involvement, the structure of the trust fund. Research found that there is much work to be	Yes

No.	Author, year	Financing mechanism/ instrument	Year of inception	Setting /Country	Methodology	Facilitators	Inhibitors	Nature/ Structure	Amount (USD) raised each year 2015-2020	Lessons from development and implementation processes	Earmarked for health (Yes/No)
							technical support for the policy.			done on the policy. The drive for the trust fund was more political than technical	
118.	111. World Health Organization [39], Health Organization [43]and World Health Organization [48]	Financial transaction tax	Not indicated	Gabon		• Easy to implement	• Protest action stating that it will restrict exchange	A 1.5% post levy on post tax of profit on currency and other transactions. Tax was paid by companies that handle remittances	Levy on currency and other financial transactions and mobile phone tax, generated $30 million in 2009	The funds are used to subsidize healthcare for the poor in Gabon. It covers more than 99% of the poor	Yes
119.	World Health Organization [39], Health Organization [43] and World Health Organization [48]	Mobile phone tax	Not indicated	Gabon	…	…	…	10% tax on mobile phone operators	Levy on currency and other financial transactions and mobile phone tax, generated $30 million in 2009	The funds are used to subsidize healthcare for the poor in Gabon	Yes

No.	Author, year	Financing mechanism/ instrument	Year of inception	Setting /Country	Methodology	Facilitators	Inhibitors	Nature/ Structure	Amount (USD) raised each year 2015-2020	Lessons from development and implementation processes	Earmarked for health (Yes/No)
120.	SAMRC [17,49]	***Social impact bond*** SAMRC HIV prevention intervention for adolescent girls and young women (AGYW)	2019	South Africa	Evaluation report	• Government payment is outcome based<br>• Donor funds implemented directly<br>• Funder and implementer have track records<br>• Less Government interference in the implementation process	• Quality of the service delivery was not optimum	***Social impact bond*** funded by Global Fund from 2019-2022<br><br>Government pays a funder who funds an institution to deliver services on behalf of the government when the outcomes have been achieved.	Not reported	• Data challenges marred the evaluation of the interventions<br>• Coverage was poor in year 1 of implementation<br>• COVID-19 lockdown/ school closures affected coverage	Yes
121.	Health Policy Project[50]	***Equity fund***	Not indicated	Nigeria	Report	• Community involvement	…	Mobilizing money from communities to provide care in the local community	…	…	…
